# Circ_0040039 May Aggravate Intervertebral Disk Degeneration by Regulating the MiR-874-3p-ESR1 Pathway

**DOI:** 10.3389/fgene.2021.656759

**Published:** 2021-06-11

**Authors:** Yongjin Li, Xuke Wang, Haiwei Xu, Guowang Li, Zhenxin Huo, Lilong Du, Kaihui Zhang, Li Shen, Hao Li, Baoshan Xu

**Affiliations:** ^1^Department of Minimally Invasive Spine Surgery, Tianjin Hospital, Tianjin, China; ^2^Graduate School, Tianjin Medical University, Tianjin, China; ^3^Tianjin Hospital, Orthopedic Research Institute, Tianjin, China; ^4^Department of Minimally Invasive Spine Surgery, Luoyang Orthopedic- Traumatological Hospital, Luoyang, China

**Keywords:** circular RNA, ESR1, apoptosis, intervertebral disk degeneration, miR-874-3p

## Abstract

The functional alteration of nucleus pulposus cells (NPCs) exerts a crucial role in the occurrence and progression of intervertebral disk degeneration (IDD). Circular RNAs and microRNAs (miRs) are critical regulators of NPC metabolic processes such as growth and apoptosis. In this study, bioinformatics tools, encompassing Gene Ontology pathway and Venn diagrams analysis, and protein–protein interaction (PPI) network construction were used to identify functional molecules related to IDD. PPI network unveiled that ESR1 was one of the most critical genes in IDD. Then, a key IDD-related circ_0040039-miR-874-3p-ESR1 interaction network was predicted and constructed. Circ_0040039 promoted miR-874-3p and repressed ESR1 expression, and miR-874-3p repressed ESR1 expression in NPCs, suggesting ESR1 might be a direct target of miR-874-3p. Functionally, circ_0040039 could enhance NPC apoptosis and inhibit NPC growth, revealing that circ_0040039 might aggravate IDD by stabilizing miR-874-3p and further upregulating the miR-874-3p-ESR1 pathway. This signaling pathway might provide a novel therapeutic strategy and targets for the diagnosis and therapy of IDD-related diseases.

## Introduction

The intervertebral disk (IVD), especially the nucleus pulposus (NP) tissue in its center, plays a crucial role in harboring complex mechanical stress and maintaining spine stability (Humzah and Soames, 1988). NP cell (NPC) degeneration is often regarded as the initiating factor of intervertebral disk degeneration (IDD). The abnormal increase in the degradation of NPC extracellular matrix (ECM) components, such as aggrecan and collagen II (Roughley, 2004; Fontana et al., 2015; Oichi et al., 2020); NPC apoptosis (Ding et al., 2013; Fontana et al., 2015; Oichi et al., 2020); and levels of proinflammatory cytokines, such as tumor necrosis factor α (TNF-α) and interleukin 1β (IL-1β) secreted by NPCs (Risbud and Shapiro, 2014; Fontana et al., 2015; Oichi et al., 2020; Wang et al., 2020), are the most important pathological characteristics during IDD. The functional changes in NPCs can trigger the loss of IVD function and further facilitate the progression of IDD (Roughley, 2004; Ding et al., 2013; Risbud and Shapiro, 2014; Fontana et al., 2015; Oichi et al., 2020; Wang et al., 2020). Thus, exploring the specific pathomechanism of IDD at the level of NPCs is of far-reaching significance.

Non-coding RNAs (ncRNAs), such as circular RNAs (circRNAs) and microRNAs (miRNAs, miRs), are vitally important regulatory elements encoded by the genome. Accumulating studies have constantly uncovered that the dysfunction of NPCs induced by proinflammatory cytokines or compression or other inducers can be recovered by differentially expressed circRNAs and miRNAs in IDD (Cheng et al., 2018; Wang et al., 2018; Xie et al., 2019; Cazzanelli and Wuertz-Kozak, 2020; Xiang et al., 2020). Mechanistically, circRNA-mediated alteration in the expression levels of miRNAs can be divided into two modes. One is the canonical sponge mechanism, in which circRNAs repress or do not affect miRNA expression (Piwecka et al., 2017; Cheng et al., 2018; Wang et al., 2018; Xie et al., 2019; Xiang et al., 2020); another is the stabilization mechanism, in which circRNAs elevate miRNA expression (Piwecka et al., 2017; Chen et al., 2019). MiRNAs are small, single-stranded, ncRNAs, which inhibit mRNA expression by inhibiting mRNA translation or inducing mRNA degradation through forming an RNA-induced silencing complex with argonaute 2 protein and directly interacting with the 3′- untranslated region (UTR) of the target mRNA (Pasquinelli, 2012; Piwecka et al., 2017; Cheng et al., 2018; Ji et al., 2018; Wang et al., 2018; Chen et al., 2019; Xie et al., 2019; Xiang et al., 2020). Circ-VMA21 (Cheng et al., 2018), circ-CIDN (Xiang et al., 2020), circ-4099 (Wang et al., 2018), circ-ERCC2 (Xie et al., 2019), and miR-141 inhibitor (Ji et al., 2018) were reported to be involved in regulating NPC apoptosis and ECM metabolism and also alleviate IDD *in vitro* and *in vivo*. However, the current treatment of IDD remains a challenge. Therefore, novel key molecules to maintain the normal physiological function of NPCs and block the pathological process of IDD are urgently needed.

In this study, IDD-related circRNA (GSE67566), miRNA (GSE63492/GSE116726), and mRNA (GSE56081) microarray datasets downloaded from the Gene Expression Omnibus (GEO) database^1^, which reposits publicly available gene expression and other functional genomic datasets, were reanalyzed (Clough and Barrett, 2016). Then, a circ_0040039-miR-874-3p-ESR1 interaction network was constructed by bioinformatics analysis, and it was confirmed that circ_0040039 could upregulate the miR-874-3p-ESR1 pathway. Finally, the overexpression of circ_0040039 was found to promote NPC degeneration.

## Materials and Methods

### Selection and Analysis of GEO Datasets

After Lan et al. (2016); Ji et al. (2018), and Wan et al. (2014) sequenced normal and degenerative NP tissues, respectively, they uploaded circRNA (GSE67566), miRNA (GSE63492/GSE116726), and mRNA (GSE56081) microarray datasets to GEO database. Detailed information for each dataset is shown in Table 1. In terms of GSE67566/GSE63492/GSE116726, the raw data were read and analyzed using the limma package in R (Ritchie et al., 2015), as well as normalized and log2-transformed. By default, the false-positive results of adjusted *P*-value were corrected using Benjamini and Hochberg false discovery rate (FDR). We identified differentially expressed miRNAs (DEMs) with the criterion of the absolute value of log2 fold change (FC) > 2 and −log_10_ (FDR) > 2 based on the analysis of GSE116726. The GSE56081 dataset was obtained from Lan et al. (2016) analytical result (FC > 2 or < −2, *P* < 0.05).

**TABLE 1:** Basic information of the microarray datasets.

**Data source(GEO)**	**Platform**	**Samples size(D/N)**	**RNA types**	**First author**	**References**
GSE67566	GPL19978	5/5	circRNA	Lan PH	Lan et al., 2016
GSE63492	GPL19449	5/5	miRNA	Lan PH	Lan et al., 2016
GSE116726	GPL20712	3/3	miRNA	Ji ML	Ji et al., 2018
GSE56081	GPL15314	5/5	mRNA	Wan ZY	Wan et al., 2014

*GEO, Gene Expression Omnibus; D, degeneration, N, normal; circRNA, circular RNA; miRNA, microRNA; mRNA, messenger RNA.*

### Venn Analysis

The upstream miRNAs of ESR1 were predicted by Targetscanhuman 7.2^2^ (Agarwal et al., 2015), mirDIP^3^ (Tokar et al., 2018), starBase^4^ (Li et al., 2014), miRTarBase^5^ (Chou et al., 2018), miRDB^6^ (Chen and Wang, 2020), and miRWalk 3.0^7^ (Dweep and Gretz, 2015) databases, and GSE63492/GSE116726 datasets. Targetscanhuman 7.2 (Agarwal et al., 2015), starBase (Li et al., 2014), miRTarBase (Chou et al., 2018), miRDB (Chen and Wang, 2020), miRWalk 3.0 (Dweep and Gretz, 2015), and miRanda^8^ (John et al., 2004), and GSE56081 dataset were used to predict miR-874-3p targets genes. Additionally, the upstream circRNAs of miR-874-3p were predicted via circbank^9^ (Liu et al., 2019), starBase (Li et al., 2014) databases, and GSE67566 dataset to select IDD-related circRNAs.

### Gene Ontology Enrichment Analyses and Protein–Protein Interaction Network Construction

Based on the miR-874-3p targets genes predicted by miRTarBase database (Chou et al., 2018), the Cytoscape software was utilized to display these genes (Otasek et al., 2019). Furthermore, Gene Ontology (GO) enrichment analyses was conducted using the Search Tool for the Retrieval of Interacting Genes (STRING)^10^ (Szklarczyk et al., 2019), and the predominant enrichment pathways were further visualized by SangerBox tool^11^. The *P* < 0.05 was regarded as statistically significant. In addition, protein–protein interaction (PPI) network was constructed, and the degree centrality of the nodes in the PPI network was speculated through the cytoHubba plug-in in Cytoscape software (Chin et al., 2014; Szklarczyk et al., 2019), of which the higher nodes degrees were considered as the hub genes (Zotenko et al., 2008).

### Acquirement, Culture, and Treatment of Human NPCs

The specific method was described in our previous study (Li et al., 2021). Human NPCs were purchased from ScienCell Research Laboratories (ScienCell, Cat. #4800, United States), which were isolated from the NP of human intervertebral disc. NPCs were cultured in Nucleus Pulposus Cell Medium (Cat. #4801, ScienCell, United States) containing 10 mL fetal bovine serum, 5 mL NPC growth supplement, and 5 mL penicillin/streptomycin solution and then were incubated at 37°C in a humidified environment with 5% CO_2_. The medium was changed every 2 days. The NPCs were passaged once a week, and well-grown NPCs were taken for subsequent experiments. To simulate the microenvironment of IDD, TNF-α, and IL-1β (10 ng/mL, Proteintech) were employed to stimulate NPCs for 24 h.

### Plasmids Construction and NPC Transfection

The miR-874-3p mimic, miR-874-3p inhibitor, and their corresponding negative controls (NCs) were obtained from JIAMAY BIOLAB (Beijing, China). The empty vector: pcDNA3.1 + Circ Mini (5,607 bp) and overexpression vector: pcDNA3.1 + Circ Mini-circ_0040039 (6,333 bp) and pcDNA3.1 + Circ Mini- circ_0004354 (5,765 bp) were designed and synthesized by HyCell Biotechnology (Wuhan, China). As for NPC transfection, culture plates were incubated at 37°C in a humidified environment with 5% CO_2_. CircRNAs plasmids or miR-874-3p mimic or inhibitor or corresponding NCs were transfected into NPCs with Lipofectamine 8000 (Beyotime, China) based on the manufacturer’s protocols. After 48-h transfection, NPCs were collected to conduct the next experiments.

### Quantitative Real-Time Reverse Transcriptase–Polymerase Chain Reaction

Total RNAs was extracted from NPCs using TRIzol Reagent (Life Technologies, Thermo Fisher Scientific, United States) according to the manufacturer’s protocols. First, 1 μg total RNA and 1 μL Geneseed^®^ Enzyme Mix (Geneseed, Guangzhou, China) were used to reverse into 20 μL complementary DNA (cDNA) through Geneseed^®^ II First Strand cDNA Synthesis Kit (Geneseed, Guangzhou, China). Next, 10 μL Geneseed^®^ quantitative polymerase chain reaction (qPCR) SYBR^®^ Green Master Mix (Geneseed, Guangzhou, China), 0.5 μL forward (F) primer (10 μM), and 0.5 μL reverse (R) primer (10 μM) were made up and used to conduct quantitative reverse transcriptase (RT)–PCR on ABI7500 system (Applied Biosystems, CA, United States). All specific primers were shown as follows: (1) GAPDH: F1: AGAAGGCTGGGGCTCATTTG, R1: GCAGGAGGCATTGCTGATGAT; (2) ESR1: F2: 5′-ACCCTCC ATGATCAGGTCCA-3′, R2: 5′-AGATCTCCACCATGCCCT CT-3′; (3) miR-874-3p: F3: ATGGTTCGTGGGCTGCCCTGGC, Com R3: GTGCAGGGT CCGAGGT, RT3: GTCGTATCCAG TGCAGGGTCCGAGGTATTCGCACTGGATA.

CGACCtcggtccc; (4) U6: F4: CTCGCTTCGGCAGCACA, R4: AACGCTTCACGA ATTTGCGT, RT4: GTCGTATC CAGTGCAGGGTCCGAGGTATTCGCACTGGATA CGACCAAATATGGAAC. Among them, GAPDH was used as circ_0040039, circ_0004354, and ESR1 control, whereas U6 was used as miR-874-3p control. Their relative expression levels were measured based on the 2^–ΔΔ^ Ct method described by Livak and Schmittgen (2001).

### Cell Counting Kit-8

The well-grown NPCs were inoculated into six-hole cell culture plates at a density of 5 × 10^5^ cells per well. Then, 200 μL diluted RNAs–Lipofectamine 8000 (Beyotime, China) complex was added to the cell wells that had been replaced with 800 μL serum-free medium. The NPCs were then cultured for 0, 1, 2, and 3 days at 37°C incubator. For Cell Counting Kit-8 (CCK8) assay, 10 μL CCK8 solution was added to each well and for incubation for another 1.5 h. The NPC growth was evaluated by CCK8 detection kit according to manufacturer’s protocols (Yeasen, Shanghai, China). The absorbance was determined at OD 450 nm. NPC growth rates were calculated based on the formula: Day*n* OD value/Day0 average OD value (same processing sample).

### Flow Cytometry

The well-grown NPCs were inoculated into 6 hole cell culture plates at a density of 5 × 10^5^ cells per well. After circ_0040039 or circ_0004354 overexpression vector were transfected into NPCs using Lipofectamine 8000 (Beyotime, China), the NPC apoptosis rates were evaluated by annexin V–APC apoptosis detection kit according to manufacturer’s protocols (keyGEN, KGA1024, China). Annexin V–APC is matched with 7-AAD to distinguish NPCs in different stages of apoptosis. The NPCs were stained with 5 μL annexin V–APC and 5 μL 7-AAD, and then the data were analyzed with FlowJo VX10 software. On the scatterplot of the bivariate flow cytometry (FCM), annexin V + /7-AAD + (Q2) represented the late apoptotic and necrotic NPCs; annexin V + /7-AAD- (Q3) represented the early apoptotic NPCs, whereas annexin V–/7-AAD− (Q4) represented living NPCs.

### Dual-Luciferase Reporter Assays

MiRanda database (John et al., 2004) was used to predict the potential binding sites of miR-874-3p with ESR1 mRNA 3′-UTR. Luciferase reporter vectors: psiCHECK2–Firefly luciferase–Renilla luciferase containing ESR1–700-bp wild-type (WT) sequences or corresponding mutant (MUT) sequences, were constructed by Geneseed Biotech Co. (Guangzhou, China). Human embryonic kidney (HEK) 293T cells were plated on 24-well plates at a density of 1 × 10^5^ cells per well. Subsequently, 1 μg vector and 100 μL miR-874-3p mimic or mimic NC were cotransfected into HEK-293T cells using 2 μL Lipofectamine 8000 (Beyotime, China). After 48-h transfection, the relative luciferase activity was measured using the Dual Luciferase Assay Kit (Promega E1910, Madison, WI, United States) according to the manufacturer’s directions. The activation degrees of the target reporter genes were calculated between different samples according to the obtained ratio of the relative light unit (RLU) value measured by Renilla luciferase divided by the RLU value measured by Firefly luciferase.

### Western Blotting Assay

The specific method was described in our previous study (Li et al., 2021). RIPA lysis buffer containing phenylmethanesulfonyl fluoride (Beyotime, Shanghai, China) was used to extract the total protein from NPCs. The protein concentrations were quantified using the Micro Bicinchoninic Acid Protein Assay kit (Beyotime, Shanghai, China). After making sodium dodecyl sulfate–polyacrylamide gel electrophoresis (SDS-PAGE) gels, the proteins were isolated through SDS-PAGE and then transferred to polyvinylidene difluoride (PVDF) membranes (Bio-Rad, CA, United States) at 350 mA for 70 min. Subsequently, the PVDF membranes were blocked by 5% non-fat milk and incubated overnight at 4°C with primary antibody, including anti-ESR1 antibody (diluted 1:1,000; Abcam, ab32063) and anti–β-actin antibody (diluted 1:5,000; Proteintech, 66009-1-Ig), followed by incubation with a secondary antibody. Phosphate-buffered saline with Tween-20 was utilized to wash the PVDF membranes. Finally, the signals were tested by BeyoECL Star Luminescence kit (Beyotime, Shanghai, China) and a chemiluminescence system (Bio-Rad, CA, United States).

### Statistical Analysis

All the experiments were performed at least three times. GraphPad Prism software 6 version was used to analyze the data. The statistical significances between the two groups were compared using unpaired Student’s *t-*test, where the differences among more than two groups were assessed using one-way analysis of variance followed by Tukey multiple-comparisons test. The *P* < 0.05 was considered as statistically significant.

## Results

### Prediction of ESR1 Was One of the Most Critical Differentially Expressed Genes in IDD

**FIGURE 1:**
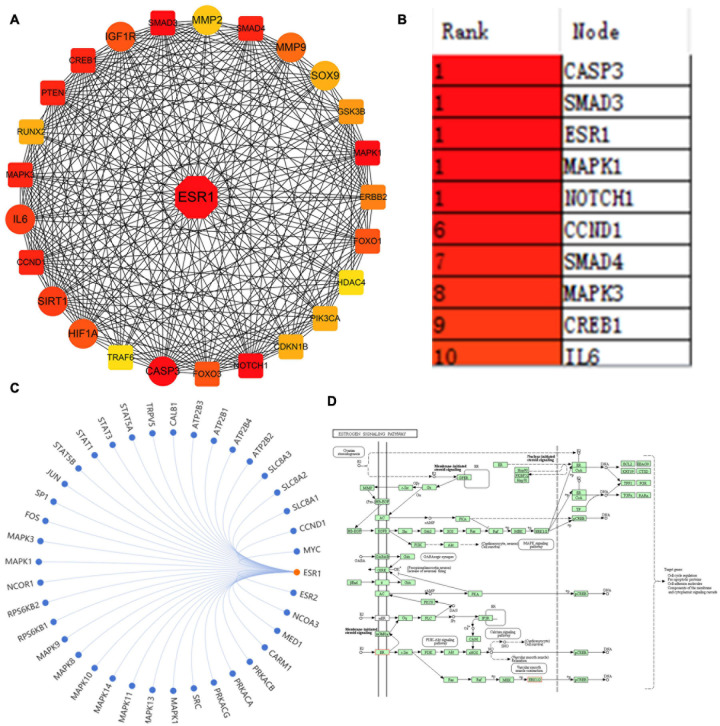
ESR1 was identified as one of the most critical genes in IDD. **(A)** PPI network showing IDD-related miRNA target genes. The line between the circle nodes indicates the interaction between the two genes. Red indicates the most key hub genes; the depth of the color is related to the association of other genes in the PPI network. **(B)** Top 10 genes in the PPI network ranked by the maximal clique centrality method, of which ESR1 ranked the highest. **(C)** Other genes regulated by ESR1 were predicted and visualized using the SangerBox tool. **(D)** Kyoto Encyclopedia of Genes and Genomes analysis of estrogen signaling pathway displayed that ESR1 might be involved in regulating various pathways.

A large number of studies have indicated the involvement of a multitude of DEMs in regulating IDD via repressing their target genes. However, which target genes were the most important remains unanswered. To address this question, the key differentially expressed genes (KDEGs) of these DEMs were summarized and are listed in Supplementary Table 1. A total of 97 different target genes were reported as IDD-related KDEGs. Given that the STRING website can provide experimental and predicted PPI information, and PPI is the most appropriate tool for studying the potential interrelationship among multiple genes, this study mapped the 97 KDEGs into the STRING website, followed by the analysis of their interaction using cytoHubba plug-in in Cytoscape software. The results unveiled that estrogen receptor α (ESR1) ranked the highest and was a hub gene, suggesting that ESR1 could regulate a series of IDD-related genes, encompassing protective factors, such as SIRT1, Sox9, HIF-1α, and IGF1R, as well as catabolic factors, such as IL-6, MMP2/9, and CASP3 (Figures 1A,B). The Kyoto Encyclopedia of Genes and Genomes analysis of the estrogen signaling pathway revealed that ESR1 might regulate the expression of related genes (Figure 1C) to mediate various signaling pathways, including canonical mitogen-activated protein kinase, PI3K-Akt, and estrogen pathways, thereby affecting cell cycle progression, growth, apoptosis, and other pathological processes (Figure 1D). Furthermore, Cai et al. (2020) demonstrated that the mRNA and protein expression levels of ESR1 were significantly decreased in patients with IDD diseases. Collectively, these results predicted that ESR1 might be one of the most KDEGs in IDD.

### Prediction of the Upstream MiRNAs of ESR1

**FIGURE 2:**
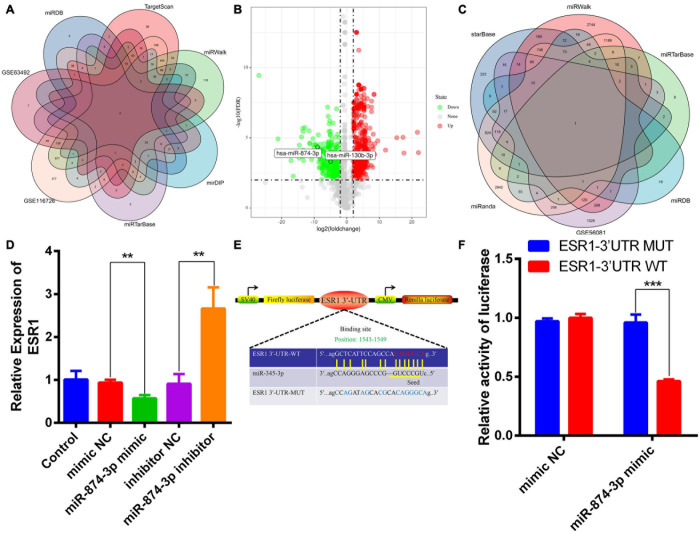
MiR-874-3p was predicted to be the upstream miRNA of ESR1. **(A)** Upstream miRNAs of ESR1 were predicted using different algorithms. **(B)** Volcano plot shows the predicted upstream miRNAs of ESR1 based on the analysis of GSE116726. Green points represent downregulated miRNAs (left side), and red points represent upregulated miRNAs (right side); miR-874-3p and miR-130b-3p are presented. **(C)** Venn analysis of miR-874-3p downstream target genes using different algorithms. **(D)** ESR1 expression level was measured in NPCs after transfected with miR-874-3p mimic or miR-874-3p inhibitor or corresponding NCs using the qRT-PCR assay. **(E)** Binding site of miR-874-3p and ESR1. **(F)** Luciferase reporter vectors carrying ESR1 WT or MUT sequences were cotransfected into HEK-293T cells with miR-874-3p mimic or mimic negative control (NC). Relative luciferase activity was detected in HEK-293T cells. Data are represented as the mean ± SD. **P < 0.01, ***P < 0.001.

MiRNAs can degrade mRNAs and inhibit their translation via directly binding to the 3′-UTR of their target mRNAs (Pasquinelli, 2012; Ji et al., 2018). The upstream miRNAs of ESR1 were predicted and analyzed by bioinformatics analysis. The datasets used in this study were obtained from human NP specimens. Two overlapped IDD-related miRNAs were predicted by merging miRDB, TargetScan, miRTarBase, mirDIP, and miRwalk databases and GSE116726/63492 datasets (Figure 2A). Volcano plots revealed two DEMs in GSE116726; the expression of miR-874-3p was lower than that of miR-130b-3p in IDD (Figure 2B). Conversely, ESR1 was predicted to the target gene of miR-874-3p by intersecting different algorithms, including the GSE56081 dataset (Figure 2C). The potential binding sites of miR-874-3p with ESR1 mRNA 3′-UTR were predicted using the miRanda database (John et al., 2004). An ESR1 fragment with WT or MUT complementary binding sites for miR-874-3p was established and inserted into psiCHECK2 luciferase reporter vectors to confirm further the interaction between miR-874-3p and ESR1 (Figure 2D). MiR-874-3p mimic significantly repressed the luciferase activity of the ESR1-WT vector, whereas such overexpression could not change the activity of the ESR1-MUT vector, revealing that miR-874-3p could directly bind to the 3′-UTR of ESR1 (Figure 2E). Moreover, miR-874-3p mimic repressed and miR-874-3p inhibitor increased the mRNA level of ESR1 (Figure 2F). Thus, miR-874-3p was determined as a key miRNA in this study.

### Bioinformatics Analysis of miR-874-3p Target Genes Predicted Using the miRTarBase Database

**FIGURE 3:**
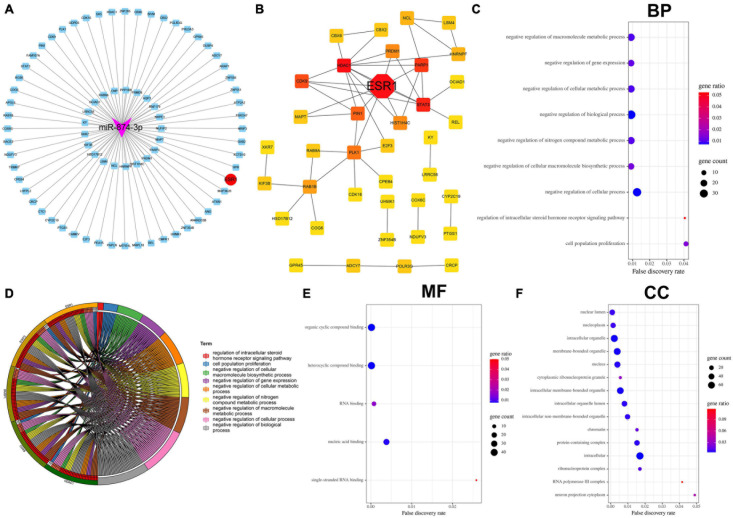
Bioinformatics analysis of miR-874-3p target genes predicted using the miRTarbase database. **(A)** Cytoscape software was used to visualize the miR-874-3p target genes, of which miR-874-3p and ESR1 were indicated with red ellipse and purple triangles, respectively. **(B)** ESR1 was identified as a key hub gene in the PPI network using the Cytoscape plug-in cytoHubba, of which ESR1 is indicted with a red diamond. **(C–F)** GO analysis of miR-874-3p target genes through the STRING website. FDR < 0.05 was regarded as statistically significant. **(C)** Bubble diagram shows the main biological process. The *x*-axis represents the FDR, the left *y*-axis represents the GO terms, and the right *y*-axis represents the gene ratio (up) and gene count (down). **(D)** GO chord diagram shows the five hub genes involved in the main biological process. The left outside of the circle represents the genes, whereas the left inside of the circle represents the FDR. **(E)** Bubble diagram shows the enrichment of molecular function. **(F)** Enrichment of the cellular component is shown by bubble diagram.

miRTarBase: The experimentally (luciferase reporter assay, Western blot, microarray, and next-generation sequencing experiments) validated miRNA–target interaction database (Chou et al., 2018) was used to predict the target genes of miR-874-3p. The 77 miR-874-3p target genes predicted using the miRTarBase database were then visualized using Cytoscape software (Figure 3A). As shown in Figure 3B, the analysis result of PPI revealed that ESR1 was the most key hub gene of the miR-874-3p target genes. Subsequently, GO functional enrichment analysis for these target genes was conducted using the STRING website, which predominantly included three aspects: biological process (BP), molecular function (MF), and cellular component (CC). The bubble diagram (Figure 3C) and GO chord diagram (Figure 3D) of the GO analysis of BP indicated that miR-874-3p might be mainly involved in regulating different signaling pathways through targeting ESR1/signal transducer and activator of transcription 3 (STAT3)/poly(ADP-ribose) polymerase 1 (PARP1)/cyclin-dependent kinase 9 (CDK9)/histone deacetylases 1 (HDAC1), encompassing negative regulation of macromolecule metabolic process, negative regulation of gene expression, negative regulation of cellular metabolic process, and cell population growth. MF included organic cyclic compound binding, heterocyclic compound binding, RNA binding, nucleic acid binding, and single-stranded RNA binding (Figure 3E). The most enriched in CC were nucleoplasm, intracellular organelle, membrane-bound organelle, cytoplasmic ribonucleoprotein granule, and protein-containing complex (Figure 3F). These results indicated that miR-874-3p might modulate NPC growth and apoptosis to mediate IDD through binding to ESR1 or other mRNAs in the cytoplasm.

### Prediction and Verification of the Upstream CircRNAs of MiR-874-3p

**FIGURE 4:**
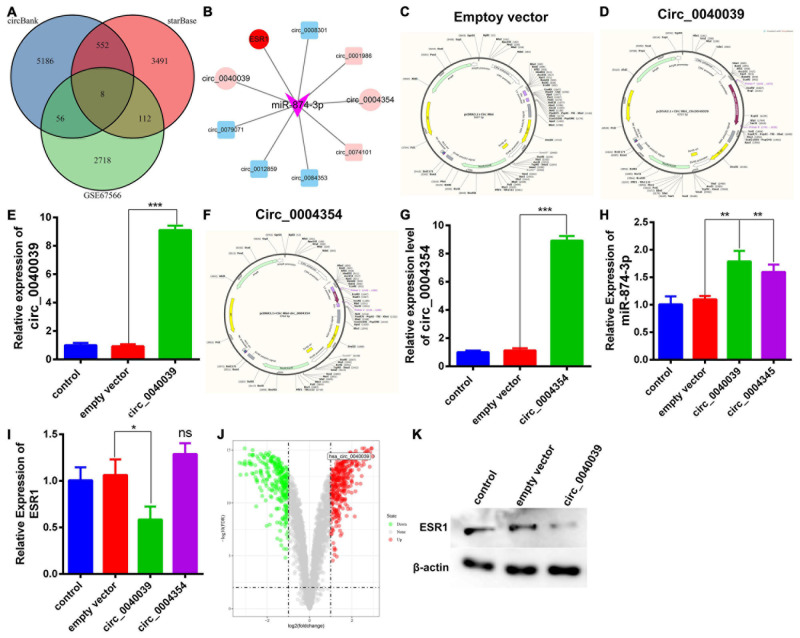
Prediction and verification of the upstream circRNAs of miR-874-3p. **(A)** Venn diagram was used to select the overlapping upstream circRNAs of miR-874-3p through the intersection of circbank and starBase databases, and the GSE67566 dataset. **(B)** CircRNAs-miR-874-3p-ESR1 interaction network was established using Cytoscape software. Light red represents upregulated circRNAs, and light green represents downregulated circRNAs. Circ_0040039, circ_0004354, and ESR1 were exhibited with a red ellipse, whereas miR-874-3p was represented by purple triangles. **(C)** CircRNA empty vector atlas. **(D)** Circ_0040039 overexpression vector atlas. **(E)** Overexpression effect of circ_0040039 was validated in NPCs using the qRT-PCR assay. **(F)** Circ_0004354 overexpression vector atlas. **(G)** qRT-PCR assay corroborated that the expression of circ_0004354 significantly increased in circ_0004354-transfected NPCs. **(H)** qRT-PCR assay confirmed that the expression level of miR-874-3p was elevated in NPCs after transfection with circ_0040039 or circ_0004354. **(I)** ESR1 expression level was measured in NPCs after transfection with circ_0040039 or circ_0004354 or corresponding NC using the qRT-PCR assay. **(J)** Volcano plot shows the predicted circ_0040039 based on the analysis of GSE67566. Green points represent downregulated circRNAs (left side), and red points represent upregulated circRNAs (right side); circ_0040039 is presented. **(K)** Western blotting assay demonstrated that circ_0040039 inhibits the protein expression level of ESR1. Data are represented as the mean ± SD. *P < 0.05, **P < 0.01, ***P < 0.001.

Accumulating evidence has uncovered that miRNAs can be adsorbed or even repressed by circRNAs via a ceRNA-dependent mechanism (Piwecka et al., 2017; Cheng et al., 2018; Wang et al., 2018; Xie et al., 2019; Xiang et al., 2020). The upstream circRNAs of miR-874-3p were predicted and analyzed to explore further the novel unidentified circRNAs affecting miR-874-3p function. Circbank is a comprehensive database of human circRNAs containing beyond 140,000 annotated circRNAs from different sources, which can be used to predict the upstream circRNAs of miRNAs (Liu et al., 2019). Eight overlapped circRNAs were predicted by intersecting starBase and circbank databases and the GSE67566 dataset (Figure 4A). Furthermore, a circRNA-miR-874-3p-ESR1 interaction network was constructed and visualized using Cytoscape software (Figure 4B). The two most upregulated IDD-related circRNAs circ_0040039 and circ_0004354, both derived from the syntrophin β2 gene, were predicted to bind to miR-874-3p together (data not shown). Thus, circ_0040039 and circ_0004354 were selected for further investigation. Subsequently, the empty vector (Figure 4C) and overexpression vector of circ_0040039 (Figure 4D) and circ_0004354 (Figure 4F) were constructed. The expression levels of circ_0040039 (Figure 4E) and circ_0004354 (Figure 4G) significantly increased after transfecting their overexpression vector into NPCs. Unexpectedly, both circ_0040039 and circ_0004354 elevated (but not repressed) the miR-874-3p expression level; the role of circ_0040039 was more significant (Figure 4H). In addition, circ_0004354 slightly elevated ESR1 expression without any statistically significant difference, whereas circ_0040039 remarkably repressed the ESR1 expression level (Figure 4I). Figure 4J displayed that circ_0040039 was the most upregulated circRNA in IDD group through the analysis of GSE67566. Furthermore, Western blotting assay demonstrated that circ_0040039 inhibited the protein expression level of ESR1 (Figure 4K). Taken together, these data suggested that circ_0040039 might regulate the miR-874-3p-ESR1 pathway via a stabilization mechanism rather than a canonical ceRNA mechanism, as previously reported (Piwecka et al., 2017; Chen et al., 2019).

### Demonstration of the Expression Levels of Circ_0040039, Circ_0004354, MiR-874-3p, and ESR1 in Proinflammatory Cytokine-Treated NPCs

**FIGURE 5:**
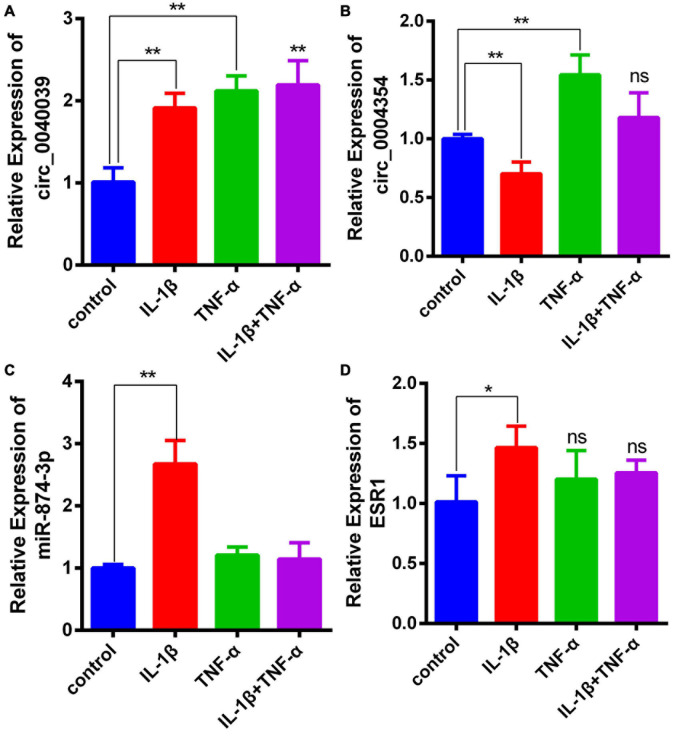
Demonstration of the expression levels of circ_0040039, circ_0004354, miR-874-3p, and ESR1 in proinflammatory cytokine–treated NPCs. **(A–D)** Expression levels of circ_0040039, circ_0004354, miR-874-3p, and ESR1 in NPCs were detected using the qRT-PCR assay in response to different proinflammatory cytokine treatments. Among these, circ_0040039, miR-874-3p, and ESR1 expression levels increased in IL-1β–treated NPCs. Data are represented as the mean ± SD. *P < 0.05, **P < 0.01.

Considering that the elevated expression of TNF-α and IL-1β is a hallmark trait during NPC degeneration (Risbud and Shapiro, 2014; Fontana et al., 2015; Oichi et al., 2020; Wang et al., 2020), many researchers used them to simulate the microenvironment of IDD *in vitro* (Cheng et al., 2018; Wang et al., 2018, 2020). Consistent with the predicted result, the expression level of circ_0040039 significantly increased in proinflammatory cytokine–treated NPCs (Figure 5A). On the contrary, circ_0004354 expression significantly decreased in IL-1β–treated NPCs (Figure 5B). The expression level of miR-874-3p significantly increased but not decreased under the treatment of IL-1β, whereas its expression was not altered in response to TNF-α alone or both TNF-α and IL-1β treatments (Figure 5C). Surprisingly, only IL-1β remarkably enhanced ESR1 mRNA expression, whereas using TNF-α and IL-1β at the same time slightly inhibited its expression without reaching statistically significant differences (Figure 5D). However, this result was inconsistent with previous study. Recently, Song et al. (2021) validated that TNF-α can inhibit ESR1 expression in NPCs. Another literature has indicated that ESR1 silencing can elevate IL-1β and TNF-α expression (Sheng et al., 2018). The difference in experimental results may be related to the state of the NPCs and the different experimental conditions. We cannot rule out that IL-1β might act as a buffer to transiently enhance miR-874-3p and ESR1 expression, thereby delaying the development of IDD. The specific mechanisms of TNF-α and IL-1β do not affect or even increase ESR1 mRNA levels in NPCs, and its biological significance needs future investigation to elucidate. Based on these results, IL-1β was used to simulate the IDD microenvironment for further investigation.

### Biofunction of Circ_0040039 in NPCs

**FIGURE 6:**
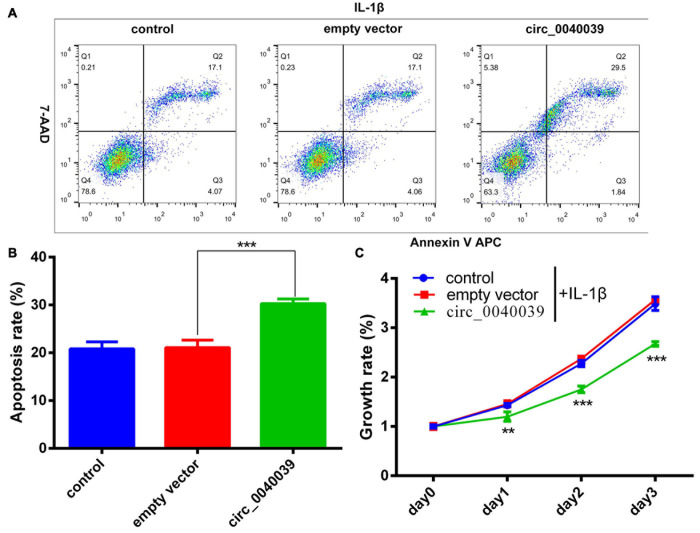
Circ_0040039 promoted NPC degeneration. **(A,B)** Circ_0040039 overexpression vector or empty vector was transfected into NPCs, and then 20 ng/mL IL-1β was added to each group to treat NPCs. **(A)** NPC apoptosis was evaluated using the flow cytometry detection assay. **(B)** Quantitative analysis of the NPC apoptosis rate. **(C)** CCK8 assay was used to detect the growth rate of NPCs. **P < 0.01, ***P < 0.001.

CCK8 and FCM detection assays were performed in circ_0040039-overexpressing NPCs to validate the biofunction of circ_0040039 in NPCs. Compared with empty vector and control, circ_0040039 significantly promoted NPC apoptosis (Figures 6A,B) and repressed NPC growth (Figure 6C) in response to 20 ng/mL IL-1β treatments. Given that circ_0040039 promoted miR-874-3p (Figure 4H) but repressed ESR1 expression (Figures 4I,J), and miR-874-3p repressed ESR1 expression in NPCs (Figure 2D), it was speculated that circ_0040039 promoted NPC degeneration possibly via activating the miR-874-3p-ESR1 signaling pathway.

## Discussion

Yang et al. (2020) summarized that estrogen can inhibit NPC apoptosis and ECM degradation by repressing proinflammatory cytokines expression and oxidative damage, as well as promoting the PI3K/Akt pathway, autophagy, and integrin expression. *Esr1* gene, which encodes the estrogen receptor α, can be activated by estrogen. Sheng and colleagues validated that ESR1 silencing or the decreased expression of ESR1 induced by miR-221 overexpression can weaken the protective effects of estrogen on IDD via inhibiting ECM synthesis, as well as elevating NPC apoptosis and IL-1β and TNF-α expression (Sheng et al., 2018). Upregulation of ESR1 was demonstrated to protect TNF-α–induced NPC degeneration through the activation of CCN5 by binding to its promoter (Song et al., 2021). Moreover, ESR1 has a negative correlation with the severity of IDD, and its mRNA and protein levels are downregulated in the NP tissues of patients with high-grade IDD compared with patients with low-grade IDD (Song et al., 2014; Cai et al., 2020). A series of studies demonstrated that the activity and biofunction of ESR1 could be regulated by circRNAs and miRNAs (Sheng et al., 2018; Cai et al., 2020; Taheri et al., 2020). The available evidence showed the interactions between ESR1 and miRNAs were implicated in the pathomechanism of IDD. For example, miR-221 (Sheng et al., 2018) and miR-203-3p (Cai et al., 2020) were reported to promote IDD via directly repressing ESR1 expression. Additionally, ESR1-associated circRNAs also have been identified in patients with cancer (Yuan et al., 2019; Xiao et al., 2020). Yuan et al. (2019) demonstrated that circ_0087378 was downregulated in patients with ER^+^ breast cancer. ESR1 was proven to inhibit circRNA-SMG1.72 expression by directly binding to the 5′ promoter region of its host gene SMG1, thereby suppressing hepatocellular carcinoma progression (Xiao et al., 2020). However, the ESR1-associated circRNAs in IDD have not been investigated. The present study found that circ_0040039 repressed whereas circ_0004354 promoted ESR1 expression. Under the stimulation of IL-1β, the expression of circ_0040039 and ESR1 was elevated in NPCs. The gain-of-function experiments revealed that circ_0040039 hindered NPC survival. Given that Lan et al. (2016) predicted and demonstrated that the expression level of circ_0040039 was remarkably upregulated in IDD, it was hypothesized that the upregulation of circ_0040039 might disrupt the normal function of IVD by inhibiting ESR1 expression and functions during IDD. The participation of circ_0040039-ESR1 pathway in regulating the ECM metabolism and the production of proinflammatory factors by NPCs, as well as the biological significance of the circ_0004354-ESR1 pathway in IDD, need further investigation.

MiR-874-3p has been implicated in regulating the apoptosis and growth of various cells. Dai et al. (2020) recently found that miR-874-3p aggravated renal podocyte apoptosis by directly inhibiting MsrB3. Leong et al. (2017) demonstrated that the activation of the miR-874-3p-PIN1 pathway promoted the apoptosis of hepatocellular carcinoma cells and repressed growth. Xia et al. (2018) also uncovered that the upregulation of miR-874-3p enhanced the apoptosis of epithelial ovarian cancer cells and inhibited growth. Huang et al. (2018) found that silencing circ_0000977 promoted the apoptosis of pancreatic ductal adenocarcinoma cells by stimulating miR-874-3p and inhibiting PLK1 expression. However, miR-874-3p has been confirmed to inhibit the apoptosis of brain tissue (Jiang et al., 2019; Xie et al., 2020) and cavernosal smooth muscle cells (Huo et al., 2020). The different roles of miR-874-3p may be related to cell state and type. This study verified that circ_0040039 and circ_0004354 promoted miR-874-3p expression, and ESR1 might be a direct target of miR-874-3p. It seemed whether miR-874-3p promoted or inhibited NPC apoptosis was not important; it might play a role as a bridge.

The cross-talk between circRNAs and miRNAs is not single. A growing body of evidence has revealed that circRNAs are widely involved in the regulation of the occurrence and progression of various chronic diseases by acting as miRNA sponges, encompassing IDD (Cheng et al., 2018; Wang et al., 2018; Xie et al., 2019; Xiang et al., 2020), osteoarthritis (Zhou et al., 2019; Chen et al., 2020), and cancers (Huang et al., 2018; Chen et al., 2019; Zhao et al., 2020), as well as cardiovascular (Garikipati et al., 2019) and neurodegenerative (Zhang et al., 2020) diseases. For instance, circ-VMA21 was demonstrated to mitigate proinflammatory cytokine–induced NPC apoptosis and ECM degradation by suppressing the miR-200c-XIAP signaling pathway (Cheng et al., 2018). Our group previously also reported that circ-FAM169A might modulate the pathological process of IDD through downregulating miR-583 (Li et al., 2021). In addition, circRNA involved in compression-induced damage of NPCs (circRNA-CIDN) (Xiang et al., 2020), circ-4099 (Wang et al., 2018), and circ-ERCC2 (Xie et al., 2019) were all corroborated to mediate the progression of IDD via negatively regulated miRNA expression. Besides adsorbing miRNA, circRNAs can also stabilize and upregulate miRNA expression. CircCSNK1G3 can positively regulate miR-181b/d expression levels to promote prostate cancer cell growth, as reported by Chen et al. (2019). Piwecka et al. (2017) found that the miR-7 expression level was downregulated and miR-7 targets were upregulated in *CDR1as*, a gene encoding circRNA Cdr1as, in knockout mouse brains. The present study also showed that circ_0040039 could enhance miR-874-3p and repress ESR1 expression levels, further supporting the existence of miRNA stabilization mechanism. However, the underlying stabilization mechanisms remain to be clarified in the future.

However, the present study also had several limitations. First, the data were obtained only from the GEO database, and hence the verification of clinical samples was lacking. Second, the study was devoid of rescue experiments and *in vivo* investigation. Third, whether circ_0040039 regulated miR-874-3p expression through a stabilization mechanism still remained unclear.

## Conclusion

Taken together, the results uncovered that circ_0040039 might inhibit ESR1 expression via upregulating miR-874-3p, thereby facilitating NPC apoptosis and inhibiting NPC growth. The findings might enhance the understanding of the pathogenesis of IDD and provide a new treatment strategy against IDD diseases in the future. The precise role and mutual regulatory mechanism of the circ_0040039-miR-874-3p-ESR1 pathway in IDD need further investigation.

## Data Availability Statement

Publicly available datasets were analyzed in this study. This data can be found here: circRNA (GSE67566), miRNA (GSE63492/GSE116726), and mRNA (GSE56081) microarray datasets.

## Author Contributions

BX and YL conceived and designed the experiments. YL, XW, HX, and GL conducted the experiments and analyzed the data. YL wrote the manuscript. ZH and KZ providedsignificant suggestions for the study. LS and HL searched the literature and collected important reference information. BX reviewed and revised the manuscript. All authors have read and approved the final version of the manuscript.

## Conflict of Interest

The authors declare that the research was conducted in the absence of any commercial or financial relationships that could be construed as a potential conflict of interest.

## Footnotes

1 http://www.ncbi.nlm.nih.gov/geo

2 http://www.targetscan.org/vert_72/

3 http://ophid.utoronto.ca/mirDIP/

4 http://starbase.sysu.edu.cn/index.php

5 http://mirtarbase.mbc.nctu.edu.tw

6 http://www.mirdb.org/miRDB/

7 https://zmf.umm.uni-heidelberg.de/

8 http://www.microrna.org/microrna/home.do

9 www.circbank.cn

10 http://www.string-db.org/

11 http://sangerbox.com/tool
